# Electroacupuncture Ameliorates Acute Myocardial Ischemic Injury and Long QT Interval in Mice through the *α*_1A_-Adrenergic Receptor: Electrophysiological, Morphological, and Molecular Evidence

**DOI:** 10.1155/2022/1984706

**Published:** 2022-06-30

**Authors:** Haiyan Zuo, Shuai Cui, Kun Wang, Xin Wu, Jie Zhou, Qiaoyu Qu, Yan Tong, Shengbing Wu, Meiqi Zhou

**Affiliations:** ^1^College of Traditional Chinese Medicine, Anhui University of Chinese Medicine, Hefei City, Anhui Province 230038, China; ^2^Research Institute of Acupuncture and Meridian, Anhui University of Chinese Medicine, Hefei City, Anhui Province 230038, China; ^3^Zhejiang Chinese Medical University, Hangzhou City, Zhejiang Province 310053, China; ^4^Anhui Maternal and Child Health Hospital, Hefei City, Anhui Province 230001, China; ^5^Xinwu District Hospital of Traditional Chinese Medicine, Wuxi City, Jiangsu Province 214028, China; ^6^Key Laboratory of Xin'an Medicine, Ministry of Education, Anhui University of Chinese Medicine, Hefei City, Anhui Province 230038, China; ^7^Bozhou Institute of Chinese Medicine, Anhui Academy of Traditional Chinese Medicine, Bozhou City, Anhui Province 236800, China

## Abstract

Acute myocardial ischemia (AMI) is a condition caused by a decrease in blood flow to the heart that can sometimes predispose to acquired long QT syndrome (LQTS), thereby resulting in sudden cardiac death. Recent evidence indicates that electroacupuncture (EA) can alleviate MI injury, but its specific mechanism remains unclear. This study was aimed at investigating the efficacy of EA, which utilizes *α*_1A_-adrenergic receptors (*α*_1A_-AR) in alleviating MI injury as well as the resulting LQTS. The AMI model was established by ligating the left anterior descending arteries (LAD) of both the wild-type and *α*_1A_ gene-knockout mice and treating them with EA for three consecutive days. A PowerLab 16 physiological recorder was used to collect the electrocardiogram (ECG) while the serum creatine kinase isoenzymes (CK-MB), lactate dehydrogenase (LDH), and norepinephrine (NE) levels in myocardial tissue were determined by using the enzyme-linked immunosorbent assay (ELISA) kit. Moreover, TTC staining was used to observe the myocardial ischemic area, while H&E and TUNEL staining determined the pathological morphology of the myocardium. Quantitative real-time PCR (qRT-PCR) was used to detect the *α*_1A_ mRNA, and Western blot was used to detect the specific proteins, such as *α*_1A_, cleaved caspase-3, Gq, PLC, p-PKC*α*, and p-hERG. Our results showed that EA could effectively reduce elevated ST-segment, shorten the extended QT interval, and reduce the serum myocardial enzyme content and the degree of pathological injury in wild mice with MI. EA can also decrease the expression of *α*_1A_-AR, PLC, p-PKC*α*, and NE content in myocardial tissues of wild mice, while those of p-hERG increased in ischemic myocardial tissue. These findings suggested that *α*_1A_-AR is involved in the development of MI as well as LQTS. Additionally, EA treatment improves the cardiac function and ischemic long QT interval and plays an important role in reducing the hERG inhibition through the *α*_1A_-AR-mediated Gq/PLC/PKC*α* pathway and myocardial apoptosis. Hence, it is suggested that *α*_1A_-AR might become a potential target for EA in treating AMI treatment of myocardial ischemia injury and acquired long QT intervals caused by MI.

## 1. Introduction

Acute myocardial ischemia (AMI) is a leading cause of death worldwide while its prevalence and mortality rate have been on a steep rise over the past few decades [1, 2]. Ventricular arrhythmias (VA) occur early in ischemia and are regarded as a common cause of sudden death in AMI [3]. Recent clinical evidence indicates that acupuncture can significantly improve the symptoms of angina pectoris in coronary heart disease patients [4, 5]. Therefore, it is of great significance to further explore the role of electroacupuncture (EA) as an effective modality to alleviate AMI injuries.

The KCNH2 gene encodes a voltage-sensitive potassium (K^+^) channel protein hERG and further mediates the fast activation delay rectifier (K^+^) channel current (IKr), which plays a major role in cardiac action potential repolarization as well as QT interval adjustment [6]. Furthermore, hERG mutation also impairs the potassium ion channels and induces cell apoptosis as well as endoplasmic reticulum (ER) stress that impacts many cardiac functions [7]. Additionally, the hERG/(IKr) channel inhibition leads to delayed ventricular repolarization and prolonged QT intervals and elicits acquired LQTS [8–11], thus triggering fatal VA [12]. Prolonged QT interval and resting membrane potential (RMP) depolarization during AMI greatly increase the risk of VA and the associated mortality [13, 14]. Corrected QT interval (QTc) based on the Bazett formula can be used as an important risk marker for patients at a higher risk of acute coronary syndrome [15]. Another study confirmed that the blockage of LAD in pigs induced AMI, which in turn prolonged the QTc interval [16]. This suggested that hERG might play a pivotal role in the pathogenesis of AMI injury and acquired LQTS. However, there is little research on the role of hERG in the therapeutic effect of EA against AMI and ischemia-induced long QT interval.

AMI is often accompanied by increased sympathetic nerve activity in conjunction with *α*_1A_-AR, which induces arrhythmia [17–19]. The *α*_1A_-AR stimulation induces ventricular fibrillation through protein kinase C (PKC) activation during AMI in mouse cardiac tissue [20]. Many previous studies [21, 22] have shown that the *α*_1A_-AR activation inhibits cardiac repolarization of hERG/(IKr) current through the PKC pathway. Based on this, it is suggested that the *α*_1A_-AR-mediated PKC pathway induces sympathetic nervous system hyperactivity that decreases hERG/(IKr) current, delays ventricular repolarization, and prolongs QT interval, thereby leading to tip torsion and ventricular fibrillation. Therefore, inhibition of *α*_1A_-AR and associated signaling pathways and recovery of hERG (K^+^) channel activity might be effective for shortening the long QT interval after AMI.

In recent years, the effectiveness and safety of acupuncture, as a component of traditional Chinese medicine (TCM), have been recognized for treating ischemic heart diseases [4, 5]. Electroacupuncture (EA) is the application of filiform needles and electrophysiological effects with repetitive frequencies that produce stable stimulation for human acupuncture points [23]. Several in vitro and in vivo experiments reported that acupuncture could improve MI by correcting the abnormalities of ventricular depolarization and repolarization in mice [24]. Moreover, autonomic nerve activity modulation as a treatment for arrhythmias has been well established in some diseases, such as long QT syndrome [25]. Our previous studies [26–28] confirmed that EA played a protective role in AMI by regulating autonomic nerve activity. Although it is suggested that acupuncture's protective effect might be due to increased expression of K^+^ channel protein in mice with myocardial infarction [29], the role of EA in improving AMI by regulating *α*_1A_-AR and potassium channels as well as shortening the long QT interval is still ambiguous.

Therefore, this study was aimed at exploring the protective effect of EA on the AMI and LQTS as well as at assessing a possible molecular basis for the role of *α*_1A_-AR in therapeutic mechanisms of EA for AMI and LQTS.

## 2. Materials and Methods

### 2.1. Experimental Animals

Adult male C57 BL/6 mice (aged 8-10 weeks; 18~22 g) were obtained from Zhejiang Hangzhou Ziyuan Experimental Animal Science and Technology Co., Ltd., China. Adult male *α*_1A_^–/–^ mice (aged 8-10 weeks; 18~22 g) of C57BL/6 genetic background were bred by Jiangsu GemPharmatech Co., Ltd., Nanjing, China, and underwent genotyping using the PCR sequence: P1258 5′CCTGCC TGAATCCATCCG3′, P1259 5′TTCCCATCAGAATACA AACATCA3′, and P1260 5′AGCTCCACATGCTCGACTGC3′. All mice were kept in environmentally controlled rooms (22 ± 2°C) with a cyclic 12 h light/12 h dark lighting schedule. All mice had free access to food and water. The experimental protocol was approved by the Animal Ethics Committee of Anhui University of Traditional Chinese Medicine (Approval ID AHUCM-2020040).

### 2.2. Instruments and Reagents

R500 small animal anesthesia machines and disposable acupuncture needles were obtained from Shenzhen Rayward Life Technology Co., Ltd. and Beijing Zhongyan Taihe Medical Instrument Co., Ltd., China, respectively. A PowerLab 16 conductive physiological recorder was from AD Instruments, Australia, while the SDZ-II EA instrument was procured from Suzhou Medical Supplies Factory, China. The following instruments were also used: RM2016 slicer (Shanghai Leica Instrument Co., Ltd., China), optical microscope (Nikon, Japan), RT-600 enzyme marker analyzer (Shenzhen Reddu, China), refrigerated centrifuge (Anhui Jiawen Instrument Equipment Co., Ltd., China), electrophoresis apparatus (BIO-RAD, USA), PS-9 rotary meter (Dalian Jingmai Technology Co., Ltd., China), and an imaging system (Shanghai Tianeng Technology Co., Ltd., China).

The following reagents were utilized: isoflurane (Shenzhen Rayward Life Technology Co., Ltd., China) and CK-MB, LDH, and NE ELISA kit from Shanghai Jianglai Biotechnology Co., Ltd., China. Additionally, the G1501 Tunel Kit was from Wuhan Servicebio, China; anti-cleaved caspase-3 antibody, *α*_1A_ antibody, anti-PLC antibody, anti-p-PKC*α* antibody, anti-p-hERG antibody, and *β*-actin antibody were purchased from Abcam (Cambridge, UK) whereas anti-Gq and sheep anti-rabbit HRP-labeled secondary antibodies were obtained from Novus Biologicals, USA, and Beijing Zhongshan Jinqiao Biotechnology Co., Ltd., China, respectively.

### 2.3. Experimental Procedure

A total of 84 mice, including 48 C57BL/6 wild-type (WT) and 36 *α*_1A_^–/–^ mice, were randomly divided into seven groups; each group had 12 mice: sham (sham operation), MI, HT7-HT5 (MI+HT7-HT5), LU9-LU8 (MI+LU9-LU8), *α*_1A_^–/–^+sham, *α*_1A_^–/–^+MI, and *α*_1A_^–/–^+MI+HT7-HT5 groups.

The AMI model was established by ligating LAD as described in our previous studies [26–28], whereas LAD was not ligated in the sham group. HT7-HT5 (WT, *α*_1A_^–/–^) and LU9-LU8 were treated with EA (Shenmen HT7, Tongli HT5, Taiyuan LU9, and Jingqu LU8) the first day after modeling, and the needle was retained for 30 min once a day for three consecutive days. EA adopted the density wave with frequency and intensity of 2 Hz/15 Hz and 1 mA, respectively, while the sham and MI groups received no treatment. Furthermore, all mice were anesthetized by inhaling 5% isoflurane (1 L/min) and were euthanized after blood sampling through orbital venous plexus after three days. After the opening of the thoracic cavity, the hearts were harvested and washed with phosphate-buffered saline (PBS), out of which four hearts were fixed in 4% paraformaldehyde and embedded in paraffin for histological analysis, while the other hearts were stored at −80°C temperature in a refrigerator for subsequent TTC staining, ELISA, Western blot, and qRT-PCR analyses.

### 2.4. ECG Analysis

As the mice were anesthetized with 2% isoflurane (1 L/min) and the limbs were fixed, needle electrodes were inserted subcutaneously into the limbs and connected to the PowerLab 16 physiological recorder. The standard II limb ECG of each group was recorded and observed followed by an analysis of the ST-segment and QTc values.

### 2.5. TTC Staining

The frozen heart was extracted and sliced into five 1 mm thick slices that were perpendicular to the long axis of the heart and were placed in the prepared 2,3,5-triphenyltetrazole chloride (TTC) staining solution. The oven temperature was maintained at 37°C for 20 min, in a dim environment. Subsequently, the MI area was white while the non-MI area appeared red. ImageJ software was used for processing images after photographic analysis. The myocardial infarct size was calculated by the MI area ratio (%) = white area/total myocardial area∗100%.

### 2.6. ELISA

The collected blood samples were allowed to stand at room temperature for one hour and then centrifuged for 15 min (4°C, 3000 r/min) to acquire the supernatant, which was further used for the detection of CK-MB and LDH markers in the mouse serum. The appropriate amount of myocardial tissue homogenate and supernatant was taken for NE detection. The ELISA test was conducted strictly according to the reagent and the kit's instructions.

### 2.7. Hematoxylin and Eosin (H&E)

After fixation with 4% paraformaldehyde for 24 h, the heart was embedded in paraffin, sliced into 4 *μ*m sections, stained with H&E, and observed under a light microscope.

### 2.8. TUNEL Assay

The TUNEL assay detection kit performed the apoptosis assay according to the kit's instructions, in which the positive cells expressed brown color. The percent of apoptotic cells was determined by counting cells in four random fields, in which the percentage of TUNEL positive cells was divided by the number of positive cells by the total number of nuclei in the field.

### 2.9. qRT-PCR

The qRT-PCR detected the expression of the *α*_1A_ gene in the myocardium. Briefly, for purification of total RNA from all the hearts, an animal total RNA rapid extraction kit was used to synthesize cDNA. Real-time PCR was amplified by a 25 *μ*L mixture of SYBR Green Mix (12.5 *μ*L), Primer Mix (0.5 *μ*L), downstream Primer (0.5 *μ*L), ddH_2_O (9.5 *μ*L), and template mixture (2 *μ*L). The GAPDH antibody was used for normalization, and Premier 5.0 software designed the primer along with the utilized primer sequence: primer F 5′CAAAGCAGGGAGTTTAGTC3′ and primer R 5′GTGAGATGGCTCTGAGTGT3′′′.

### 2.10. Western Blot Analysis

The myocardial tissue was cut into small fragments, and the lysate was added to the proportion of 150–250 *μ*L lysate per 20 mg of tissue. The purified protein lysate was extracted, and the protein samples were quantified with the BCA protein quantitative reagent. After preparing separation and concentrated gels, an equal amount of total protein was added to each well for electrophoresis and transferred to PVDF membranes which were later immersed in 5% skim milk for one hour and incubated with primary antibodies *α*_1A_ (1 : 1000), anti-Gq (1 : 1000), anti-PLC (1 : 500), anti-p-PKC*α* (1 : 1000), anti-p-hERG (1 : 10 000), anti-cleaved caspase-3 (1 : 500), or *β*-actin (1 : 5000) at 4°C overnight. Then, the membrane was incubated with a diluted HRP-labeled secondary antibody (1 : 10000) at 37°C for one hour. The protein bands were observed by using ECL luminescence solution and analyzed by ImageJ software.

### 2.11. Statistical Analysis

All experimental data were expressed by mean ± SD and analyzed by SPSS 21.0 software. The histogram was drawn by GraphPad Prism 8.0. The statistical differences between the groups were evaluated by one-way variance analysis (ANOVA), while the post hoc least significant difference (LSD) multiple comparison tests and Student's *t*-test were used for comparison between the two groups. The difference was considered statistically significant at ^∗^*p* < 0.05 and ^∗∗^*p* < 0.01.

## 3. Results

### 3.1. MI Injury and Long QTc Interval Were Related to an Increased Expression of *α*_1A_-AR in the Myocardium

**Figure 1:**
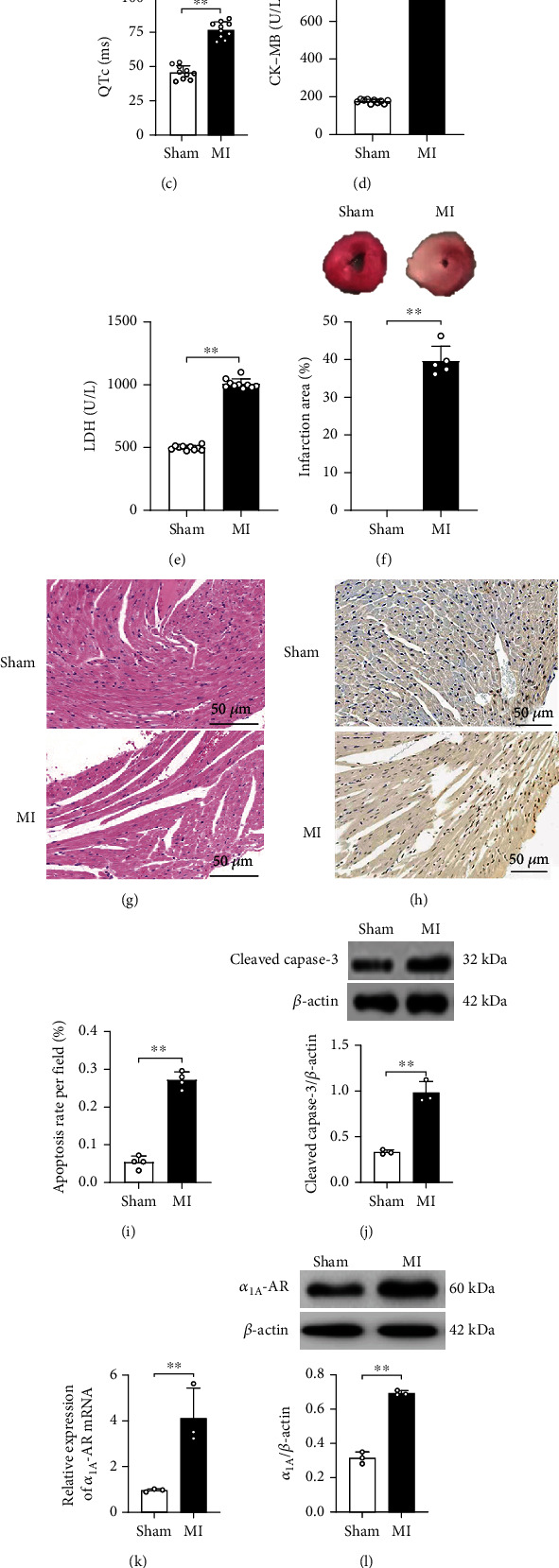
MI injury and long QTc interval were associated with an increased *α*_1A_-AR expression in the mouse myocardial tissue. ECG showed ST-segment displacement (a, b) and QTc (a, c) after 3 days in MI mice. (d, e) The content of serum myocardial mouse enzymes with MI. (f) The myocardial infarct area was detected by TTC staining. The top figure shows the representative image of TTC staining, and the bottom figure shows the quantitative results of the infarct area. (g) Representative H&E staining of myocardial tissue, magnification: 200x scale bar: 50 *μ*m. (h, i) Representative picture of TUNEL staining and quantitative analysis of myocardial analysis. (j) Western blot images and quantitative analysis of cleaved caspase-3 expression. (k) qRT-PCR quantitatively shows *α*_1A_ mRNA expression in myocardial tissue. (l) Protein of *α*_1A_ was analyzed by Western blot. Relative protein expression of *α*_1A_ and *β*-actin was used as a control for protein loading. (m, n) Correlation analysis of *α*_1A_ protein with ST-segment displacement and QTc. Data were presented as mean ± SD. Statistical significance was determined by Student's *t*-test. ^∗∗^*p* < 0.01.

To examine the role of *α*_1A_-AR in MI and long QTc induced by MI injury, the association between MI and QTc lengthening as well as altered *α*_1A_ expression in the mouse heart was determined. As shown in Figures 1(a)–1(c), ECG showed ST-segment elevation and QTc interval prolongation in MI mice, while ELISA revealed that the serum CK-MB and LDH contents in MI mice increased significantly (Figures 1(d) and 1(e)). A significant increase in the myocardial ischemic area in the MI group was detected by quantitative TTC staining (Figure 1(f)). H&E staining results showed that the sham group had no abnormal myocardial morphology, whereas the MI group expressed myocardial fiber fracture and inflammatory cell infiltration in the interstitium (Figure 1(g)). TUNEL staining depicted that the apoptosis rate of myocardial cells in the MI group was significantly higher than that in the sham group (Figures 1(h) and 1(i)). The Western blot assay revealed that cleaved caspase-3 protein expression was significantly higher in the MI group when compared with the sham group (Figure 1(j)). In addition, qRT-PCR showed that the expression of *α*_1A_ mRNA in MI mice was significantly increased after the upregulation of *α*_1A_ protein (Figures 1(k) and 1(l)). Lastly, the correlation analysis stated that *α*_1A_ had a positive correlation with MI injury and long QTc (Figures 1(m) and 1(n)). These results suggest that MI and long QTc induced by MI might be associated with abnormal elevation of *α*_1A_.

### 3.2. EA at HT7-HT5 Can Alleviate MI Injury and Long QTc and Decrease the Expression of *α*_1A_-AR in MI Mice

**Figure 2:**
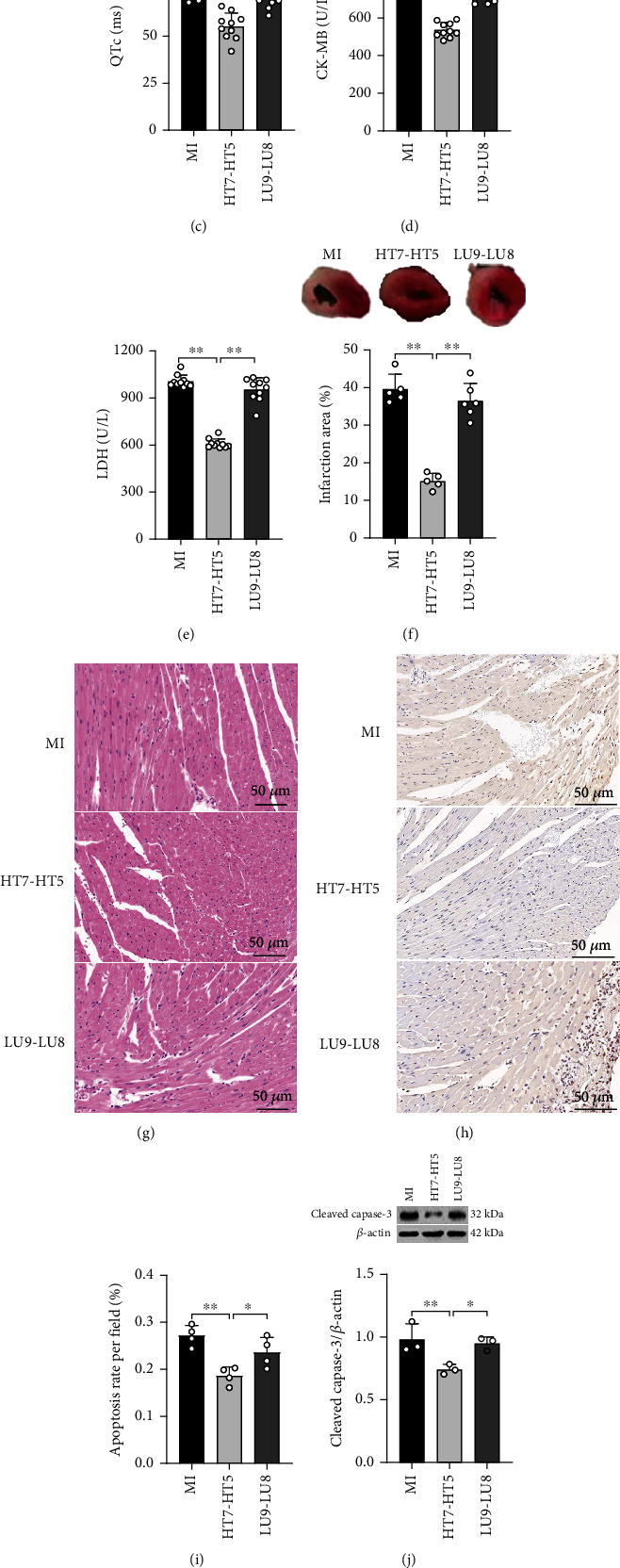
EA alleviates MI injury, shortens LQT interval, and reduces the expression of *α*_1A_-AR in myocardial tissue. ECG showed ST-segment displacement (a, b) and QTc (a, c) after 3 days of EA at different acupuncture points in MI mice. (d, e) The content of serum CK-MB and LDH. (f) TTC staining: the top picture shows the TTC staining representative image, and the bottom figure showed the quantitative result of the infarct area. (g) Representative H&E staining of myocardial tissue. Magnifications: 200x; scale bar: 50 *μ*m. (h, i) Representative picture of TUNEL staining and quantification of cardiomyocyte apoptosis. (j) Representative Western blot images showed the protein expression of cleaved caspase-3 in myocardial tissue. (k) Expression of *α*_1A_ mRNA in mouse myocardial tissue. (l) The protein expression of *α*_1A_ in myocardial tissue. Data were presented as mean ± SD. One-way ANOVA with the post hoc LSD multiple comparison test. ^∗^*p* < 0.05, ^∗∗^*p* < 0.01.

ECG, histopathological, and Western blot assessments were conducted after EA intervention for 3 days to observe the effects of EA on MI injury, long QTc interval, and *α*_1A_ expression. As shown in (Figures 2(a)–2(c), ECG showed that the ST segment of the HT7-HT5 group decreased along with the shortening of the QTc interval as compared with the MI and LU9-LU8 groups, whereas ELISA showed that the contents of serum CK-MB and LDH in the HT7-HT5 group decreased significantly (Figures 2(d) and 2(e)). TTC staining displayed that the MI area in the HT7-HT5 group was significantly reduced (Figure 2(f)). H&E staining showed broken myocardial fibers along with abundant interstitial infiltration of inflammatory cells in the MI group, whereas myocardial fibers in the HT7-HT5 group were organized and had decreased interstitial inflammatory cell infiltration (Figure 2(g)). TUNEL staining results showed that the apoptosis ratio of myocardial cells in the HT7-HT5 group was significantly reduced when compared to MI and LU9-LU8 groups (Figures 2(h) and 2(i)). The Western blot assay described that the cleaved caspase-3 protein in the HT7-HT5 group was significantly lower than that in the MI and LU9-LU8 groups (Figure 2(j)). In addition, the expression of *α*_1A_ mRNA and *α*_1A_ protein was significantly decreased in the HT7-HT5 group when compared with the MI and LU9-LU8 groups (Figures 2(k) and 2(l)). Hence, these results suggest that EA at the HT7-HT5 segment can improve MI injury and long QTc interval than that at the LU9-LU8 segment, and this effect may be related to a decreased *α*_1A_-AR expression in myocardial tissue.

### 3.3. The Cardioprotective Effect of EA at HT7-HT5 Was Significantly Reduced in the *α*_1A_^–/–^ Mice

**Figure 3:**
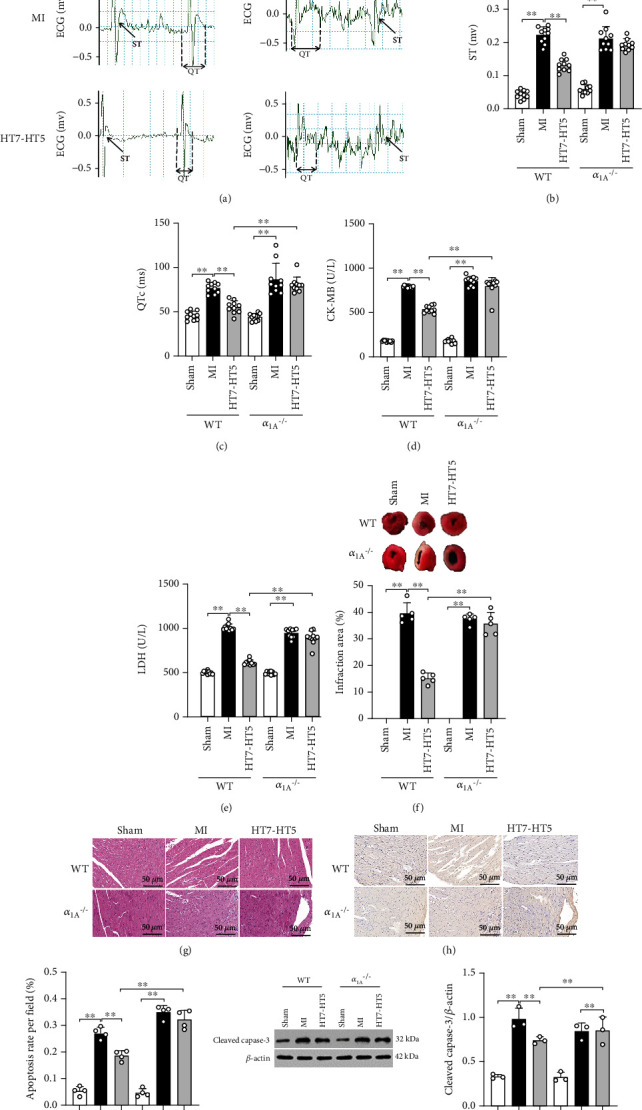
The effect of EA on MI injury and long QTc was blocked in *α*_1A_^–/–^ mice with MI. (a–c) ECG detection showed ST-segment and QTc interval in mice. (d, e) The content of serum CK-MB and LDH. (f) TTC staining: the top picture shows the TTC staining representative image, and the bottom figure shows the quantitative result of the infarct area. (g) Representative H&E staining of myocardial tissue. Magnification: 200x; scale bar: 50 *μ*m. (h, i) TUNEL staining. The representative picture indicated the apoptosis in cardiomyocytes. The figure shows the quantification of cardiomyocyte apoptosis. Magnification: 200x; scale bar: 50 *μ*m. (j) Protein expression levels of cleaved caspase-3. Data presented are mean ± SD. One-way ANOVA with the post hoc LSD multiple comparison test. ^∗^*p* < 0.05, ^∗∗^*p* < 0.01.

In order to verify whether *α*_1A_ deficiency affects the effect of EA on MI injury and long QTc, we prepared *α*_1A_^**–/–**^ mice. As shown in ECG (Figures 3(a)–3(c)), in WT and *α*_1A_^–/–^ mice, ST-segment elevation and QTc prolongation in the MI group and the ST-segment and QTc interval of mice in the HT7-HT5 group decreased when compared with the MI group in WT mice. When compared with WT mice, ST-segment and QTc interval of *α*_1A_^–/–^ mice did not change significantly when EA was given at HT7-HT5 after MI. The contents of serum CK-MB and LDH significantly decreased after EA at HT7-HT5 under conditions of MI in WT mice (Figures 3(d) and 3(e)). Under the same conditions, the contents of serum CK-MB and LDH in the HT7-HT5 group were not significantly improved in *α*_1A_^–/–^ mice (Figures 3(d) and 3(e)). We measured the percentage of the MI area with TTC staining. After MI, the percentage of the ischemic area in *α*_1A_^–/–^ mice increased, and the results were similar to those of WT mice (Figure 3(f)). Our results showed that the MI area in WT mice significantly reduced after EA inception at the HT7-HT5 segment, which was better than that in *α*_1A_^–/–^ mice (Figure 3(f)). Next, H&E staining was used to detect myocardial tissue damage. The arrangement of myocardial fibers was organized, and the infiltration of interstitial inflammatory cells decreased after EA at the HT7-HT5 segment under MI conditions in WT mice (Figure 3(g)). As compared to the WT mice, the degree of myocardial tissue injury in *α*_1A_^–/–^ mice was not significantly reduced under the same conditions (Figure 3(g)). Furthermore, TUNEL staining showed that the proportion of cardiomyocyte apoptosis in *α*_1A_^–/–^ mice was increased (Figures 3(h) and 3(i)), while the expression of the cleaved caspase-3 protein was significantly increased after myocardial infarction, similar to that in WT mice (Figure 3(j)). After EA, the percentage of myocardial cell apoptosis and cleaved caspase-3 protein expression was significantly reduced in WT mice, which was better than that in *α*_1A_^–/–^ mice (Figures 3(h)–3(j)). Therefore, *α*_1A_^–/–^ mice reversed the protective effect of EA on MI injury and long QTc in mice. These results suggest the potential role of *α*_1A_-AR in EA therapy for MI.

### 3.4. NE in Myocardial Tissue in Mice with MI after EA

**Figure 4:**
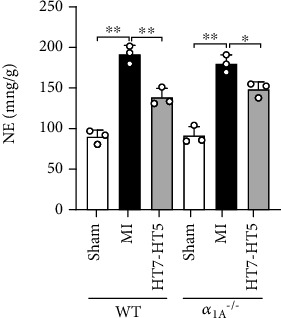
NE content in myocardial tissue. Data presented are mean ± SD. One-way ANOVA with the post hoc LSD multiple comparison test. ^∗^*p* < 0.05, ^∗∗^*p* < 0.01.

To further confirm the role of *α*_1A_, we used ELISA to detect the NE content in WT and *α*_1A_^–/–^ mice. As shown in Figure 4, in WT and *α*_1A_^–/–^ mice, NE content in myocardial tissue in the MI group significantly increased and was reduced after EA therapy. Our result showed that *α*_1A_ deficiency did not affect the release of NE. It is suggested that *α*_1A_-AR may be a key factor in EA for MI.

### 3.5. *α*_1A_^–/–^ Reversed the Effect of EA on the Gq/PLC/PKC*α*/hERG Pathway

**Figure 5:**
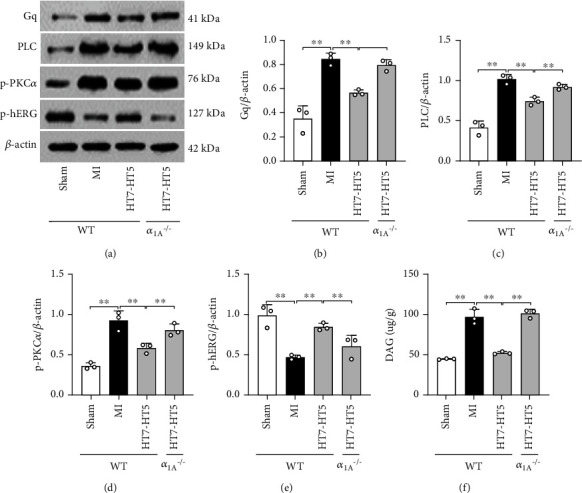
*α*_1A_^–/–^ reverses the regulation of EA on the Gq/PLC/PKC*α* pathway and hERG. (a–e) Western blot images showed the protein levels of PLC and the phosphorylation levels of p-PKC*α* and p-hERG in the mouse myocardium. (f) ELISA showed the DAG content in the mouse myocardium. Data are expressed as mean ± SD. One-way ANOVA with the post hoc LSD multiple comparison test. ^∗∗^*p* < 0.01.

In order to explore the mechanism of *α*_1A_ in inhibiting myocardial infarction injury and long QTc interval after EA, we observed the protein expression levels of Gq, PLC, PKC*α*, and hERG proteins in myocardial tissue of WT and *α*_1A_^–/–^ mice. As seen in Figure 5, Western blot analysis showed that compared with the sham group, the expressions of Gq, PLC, and p-PKC*α* in myocardial tissue of MI mice significantly increased (Figures 5(a)–5(d)), while the p-hERG expression decreased (Figure 5(e)). The protein expression of Gq, PLC, and PKC*α* decreased in WT mice after EA, while the p-hERG expression was upregulated. These effects were significantly reduced in *α*_1A_^–/–^ mice. These results suggest that EA inhibits hERG through *α*_1A_-AR, thereby reducing the activities of Gq, PLC, and PKC*α* to increase potassium current, accelerate cardiac repolarization, alleviate MI injury, and shorten the long QTc interval. *α*_1A_^–/–^ reversed the regulatory effect of EA on Gq/PLC/PKC*α* and hERG in MI mice.

## 4. Discussion

Our previous study showed that EA can alleviate MI injury in rats by inhibiting sympathetic hyperexcitation [26, 27]. In this study, EA displayed a protective effect on myocardial tissue morphology and cardiac function, as well as decreasing the long QT interval in MI mice. These beneficial effects of EA were associated with downregulation and upregulation of *α*_1A_-AR and hERG in myocardial tissue, respectively. EA affects the Gq-PLC-PKC*α* pathway by reducing the hERG channel inhibition by obstructing NE release from sympathetic preganglia. Furthermore, as the *α*_1A_-AR stimulation is reduced, the long QT interval gets shortened, and the myocardial injury diminishes. It was observed that *α*_1A_-AR knockout reversed the protective effect of EA on myocardial and long QT intervals in MI mice.

According to a few previous studies, EA can inhibit sympathetic activity and reduce MI injury [27, 28, 30]. It was also discovered that EA activity at the HT7-HT5 point can alleviate MI injury in rats by reducing ST-segment amplitude, serum myocardial enzyme levels, myocardial NE content, and MI area, thus suggesting that EA on hand shaoyin heart meridian points can alleviate MI injury by inhibiting sympathetic nerve excitability [26]. These findings were consistent with our study results. The protective effect of EA on MI is mediated by inhibition of sympathetic activity, and *α*_1A_-AR may be involved. A previous study had shown that *α*_1A_-AR blockage is beneficial during MI and is consistent with the degree of protection obtained by activating *α*_1_-AR before MI [31]. Therefore, EA might reduce the stimulation of *α*_1A_-AR and downstream signal pathways by reducing the NE content of myocardial tissue and the resultant MI injury. However, *α*_1A_-AR knockout significantly eliminated the protective effect of EA on MI.

Recent evidence indicates that arrhythmia induced by MI is related to abnormal innervation of cardiac sympathetic nerves [32, 33]. *α*_1A_-AR is one of the G protein-coupled receptors (GPCRs), which belongs to the *α*_1_-AR subtype and plays an important role in cardiac physiology and pathology. The *α*_1A_ activation induced by high sympathetic nerve activity might be an important cause of arrhythmia after MI. A study found that ventricular tachycardia (VT) could be repeatedly stimulated by injecting the *α*_1_ adrenergic agonist phenylephrine into mice [34]. Furthermore, the effect of catecholamine on isolated mouse heart ischemia-induced arrhythmias is mediated mainly by WB 4101-sensitive *α*_1A_-AR activation [35]. Since catecholamines induce *α*_1_-AR activation during MI, the increased expression of *α*_1A_-AR is closely related to arrhythmia after MI [18, 20, 36, 37]. MI-induced acquired LQTS, characterized by significant changes in QT interval length on ECG, is a prime cause of VA and sudden cardiac death in patients. Corrected QT (QTc) is generally used as an index to predict VA after MI [15, 38, 39]. In our study, it was observed that the QTc value of mice after MI was significantly longer than before, which was consistent with the previous studies [16]. Recent studies suggest that *α*_1A_-AR activation reduces cardiac repolarization K^+^ current, leading to acquired LQT [40], while the *α*_1_-AR blockers display antiarrhythmic effects in LQT2 animal models, thereby suggesting that *α*_1A_-AR might prove to be an effective therapeutic target for acquired LQTS [41]. Pytka et al. suggested that effective *α*_1A_-AR blockage is essential to reduce adrenaline-induced arrhythmias [42]. Our results reported that *α*_1A_ expression increased in mouse myocardial tissue after MI and was proportional to the prolonged QTc value. EA at the HT7-HT5 point can effectively reduce the prolonged QTc value and *α*_1A_ expression of the myocardium in mice when compared to EA at the LU9-LU8 point. However, these effects are reversed in *α*_1A_^–/–^ mice. Therefore, our results suggest that EA might shorten MI-induced long QT intervals by decreasing *α*_1A_-AR expression.

The pathogenesis of acquired long LQTS involves hERG channels and fast delay rectifying K^+^ current (IKr) [43–45]. hERG is voltage-sensitive potassium (K^+^) channel protein encoded by the KCNH2 gene, which mediates fast delayed rectifying K^+^ current [46]. Fast delayed rectification K^+^ current (IKr) plays a key role in cardiac repolarization, and reduced IKr can lead to arrhythmias [6, 47]. The decrease in hERG activity caused by gene mutation, drug inhibition, or MI can reduce IKr and slow ventricular repolarization, thus prolonging QT interval and leading to LQTS [8, 48]. Therefore, hERG activation may become a new pathway for treating acquired LQTS. Activation of *α*_1A_-AR has been reported to inhibit cardiac repolarization of hERG/I(Kr) potassium current through the PKC pathway [21, 22]. Liu et al. further demonstrated that PKC*α* mediated the inhibitory effect of *α*_1A_ on I(Kr) through the whole-cell patch-clamp technique [40]. However, *α*_1A_-AR, in conjunction with Gq, activates downstream molecules and plays an important role in cardiac diseases such as cardiac hypertrophy and arrhythmia [49–52]. It not only regulates the Ca^2+^ processing of cardiomyocytes but also directly changes the electrophysiological characteristics of the heart [53], which helps in transmembrane signal transduction of *α*_1A_-AR [54]. The Gq signal activation triggers PLC activity and hydrolyzes membrane-bound phosphatidylinositol-4,5-diphosphate (PIP2) to form diacylglycerol (DAG); DAG activates PKC and inositol 1,4,5 triphosphate (IP3) and regulates the activity of related ions [55]. PLC-mediated PKC activation has been reported to be associated with the inhibition of time-dependent K^+^ current (IKH) by *α*_1A_-AR stimulation in atrial fibrillation [56]. Our results showed that Gq, PLC, and p-PKC*α* protein expressions increased while p-hERG expression decreased with an increased *α*_1A_-AR expression in MI mice. EA can inhibit *α*_1A_-AR and its downstream signals and upregulate hERG expression to increase cardiac repolarization IKr potassium current and shorten the long QT interval. When *α*_1A_-AR was knocked out, the signal regulation of EA weakened, leading to decreased hERG protein expression and the long QTc interval, thus leading to insufficient improvement in MI. This suggests that the improvement of long QT interval by EA treatment depends on the reduction of inhibition of the hERG potassium channel by *α*_1A_-AR and downstream Gq-PLC-PKCa signal. Our results are similar to the results from previous studies, which suggested that in HEK293 cells, the intracellular pathways linking *α*_1_-AR to hERG include the activation of Gq, PLC, PIP2, and PKC [22], and PKC*α* mediates hERG inhibition when *α*_1A_-AR is activated [40].

It has been reported that hERG mutations inactivate cell membrane potassium channels and affect the imbalance of Ca^2+^ and Na^+^ both inside and outside the cells, leading to cell apoptosis and affecting cardiac structural abnormalities [7]. It has also been found that LQTS mutation can induce apoptosis by activating endoplasmic reticulum stress (ERS). Our results showed that the *α*_1A_ protein expression was increased, whereas the hERG protein expression decreased in MI mice, and the apoptosis rate was significantly higher than that in the sham group. Furthermore, due to inhibition of *α*_1A_ by EA, hERG was activated, leading to a reduced expression of cleaved caspase-3 and myocardial cell apoptosis. Previous studies have reported that *α*_1A_^–/–^ in mice leads to increased apoptosis and fibrosis of uninfarcted distal cardiomyocytes [57, 58], thus suggesting that *α*_1A_-AR is involved in cardiomyocyte survival. In contrast, sustained activation of *α*_1A_-AR induced by catecholamines does not benefit the cardiac functions after MI [20, 59, 60]. Taken together, this study further elucidates the protective mechanism of EA on MI and suggests that *α*_1A_-AR may be an important molecular target for EA treatment and prevention of MI-induced acquired LQTS and can provide a theoretical basis and experimental support for further studies on EA treatment and rehabilitation of such diseases.

In this study, *α*_1A_ gene-knockout mice were used to test the effect of EA on MI injury and the relation of LQT interval to *α*_1A_-AR expression. The potential limitation of this study is that there was no drug-positive or a negative control group. Considering that EA was only used as in vivo stimulation, the observation of IKr current in mouse cardiomyocytes was not carried out. In future studies, drug control trials and whole-cell patch-clamp techniques should be considered for positive patient outcomes.

## 5. Conclusion

**Figure 6:**
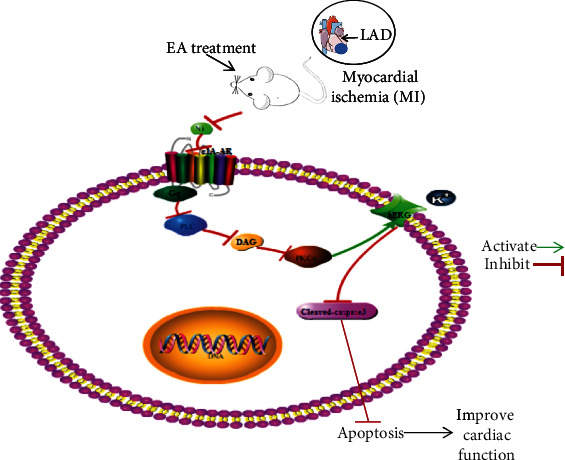
The mechanism of *α*_1A_-AR-mediated EA in improving MI injury and long QTc interval.

In conclusion, our results suggest that *α*_1A_-AR plays an important role in the EA therapy for MI. EA may upregulate hERG expression by inhibiting the Gq/PLC/PKC*α* pathway mediated by *α*_1A_-AR, shortening the MI-induced LQT interval, reducing the occurrence of ventricular arrhythmias, and ultimately playing a protective role in cardiac function (Figure 6). Our findings further enrich the mechanism of EA treatment for MI injury and acquired LQTs caused by ischemia and provide potential therapeutic modalities for clinical intervention and rehabilitation of such diseases.

## Data Availability

The data used to support the findings of this study are available from the corresponding author upon request.
